# The association between sleep disturbances and suicidal behaviors in patients with psychiatric diagnoses: a systematic review and meta-analysis

**DOI:** 10.1186/2046-4053-3-18

**Published:** 2014-02-25

**Authors:** Shaista Malik, Amrit Kanwar, Leslie A Sim, Larry J Prokop, Zhen Wang, Khalid Benkhadra, Mohammad Hassan Murad

**Affiliations:** 1 Division of Preventive Medicine, Mayo Clinic, 200 First Street SW, Rochester, MN 55905, USA; 2 Fairview Clinics, Brooklyn Park, MN, USA; 3 University of Wisconsin-Madison, Madison, WI 53706, USA; 4 Department of Psychiatry & Psychology, Mayo Clinic, Rochester, USA; 5 Mayo Clinic Libraries, Mayo Clinic, Rochester, USA; 6 Division of Health Care Policy and Research, Department of Health Sciences Research, Mayo Clinic, Rochester, USA; 7 Mayo Clinic Robert D. and Patricia E. Kern Center for the Science of Health Care Delivery, Rochester, USA; 8 Knowledge and Evaluation Research Unit, Mayo Clinic, Rochester, USA

**Keywords:** Sleep disturbances, Suicidal behaviors, Systematic reviews, Meta-analysis

## Abstract

**Background:**

Identifying patients with increased risk of suicidal behaviors is a constant challenge and concern for clinicians caring for patients with psychiatric conditions. We conducted a systematic review to assess the association between suicidal behaviors and sleep disturbances in psychiatric patients.

**Methods:**

A systematic literature search of Ovid Medline In-Process & Other Non-Indexed Citations, Ovid MEDLINE, Ovid EMBASE, Ovid PsycInfo, Ovid Cochrane Database of Systematic Reviews, Ovid Cochrane Central Register of Controlled Trials, and Scopus was conducted using earliest inclusive dates to 28 June 2013. Eligible studies were comparative observational studies that reported sleep disturbances in psychiatric patients and the outcome of interest (any type of suicidal behaviors). Pairs of reviewers extracted descriptive data, study quality, and outcomes. Odds ratios (OR) and 95% confidence intervals (CI) were pooled across studies using the random-effects model. Newcastle-Ottawa scale was used to critically appraise study quality.

**Results:**

Nineteen studies met the inclusion criteria. Compared to those without sleep disturbances, patients with psychiatric diagnoses and co-morbid sleep disturbances were significantly more likely to report suicidal behaviors (OR = 1.99, 95% CI 1.72, 2.30, *P* <0.001). The association was also demonstrated across several psychiatric conditions including depression (OR = 3.05, 95% CI 2.07, 4.48, *P* <0.001), post-traumatic stress disorder (PTSD) (OR = 2.56, 95% CI 1.91, 3.43, *P* <0.001), panic disorder (OR = 3.22, 95% CI 1.09, 9.45, *P* = 0.03), and schizophrenia (OR = 12.66, 95% CI 1.40, 114.44, *P* = 0.02). In subgroup analysis based on the type of sleep disorder, we also found suicidal behavior to be significantly associated with the presence of insomnia, parasomnias, and sleep-related breathing disorders, but not hypersomnias.

**Conclusions:**

This systematic review and meta-analysis suggests that in patients with psychiatric diagnoses, sleep disturbances are associated with the increased risk of suicidal behaviors.

## Background

Psychiatric patients are thought to be at high risk of suicidal behaviors. However, clinicians face challenges of identifying patients at risk [1]. The literature suggests that the severity of psychiatric illness is not always predictive of suicide attempts [2]; and the sensitivity and specificity of risk factors, including gender, prior suicide attempts, and suicidal ideations, remain low in predicting future suicide [3]. There is a great need to find additional risk factors. Given that sleep disturbances are fairly common in psychiatric patients, it is suggested that sleep disturbances may constitute a modifiable risk factor for suicidal behaviors [4,5].

Direct relationship between sleep disturbances and suicide has been evaluated in multiple studies. A recent systematic review and meta-analysis of 39 studies found patients with sleep problems had significantly increased risk of suicidal ideations, suicide attempts, and completed suicides [6]. That study focused on the general population although it reported that depression did not moderate the association between sleep and suicide. However, other studies found significant associations between suicides and sleep disturbances among psychiatric patients. A study of 954 patients with affective disorders found that global insomnia was significantly associated with attempted suicide [1]. In another study, both insomnia and hypersomnia had prognostic significance in predicting suicide among patients with major depression [7]. Moreover, in an epidemiologic prospective study of 1,231 psychiatric outpatients, nocturnal sleep disturbances, particularly frequent insomnia and recurrent nightmares, were independently associated with enhanced suicidal risk [8].

Considering the lack of a systematic review that focuses on the effect of sleep on suicide risk in patients with psychiatric conditions and the conflicting results from the literature, we conducted this study to assess the association between suicidal behaviors and sleep disturbances in psychiatric patients.

## Methods

A review protocol was developed at the beginning of this study. The reporting of this systematic review is in accordance with the Preferred Reporting Items for Systematic Reviews and Meta-analyses (PRISMA) statement [9].

### Inclusion and exclusion criteria

Eligible studies were comparative observational studies that reported sleep disturbances in psychiatric patients and the outcome of interest (any type of suicidal behaviors). We used the original study’s definition of sleep disturbances, which ranged from snoring, nightmares, hypersomnia insomnia, sleep panic attacks, and sleep deprivation. With respect to suicidal behaviors, we include suicide ideations, suicide attempts, completed suicides, and any other suicidal behaviors defined by the original studies. Studies were excluded if they did not report outcomes for psychiatric patients or did not provide sufficient data to quantitatively estimate the association between suicidal behaviors and sleep disturbances. We also excluded publications without original data (clinical reviews, editorials, letters, or erratum). No language or country restrictions were used.

### Data sources and search strategy

An expert reference librarian and study authors with expertise in conducting systematic reviews developed the search strategy. A comprehensive search of databases from each database’s earliest inclusive dates to 26 June 2013 was conducted. The databases included Ovid Medline In-Process & Other Non-Indexed Citations, Ovid MEDLINE, Ovid EMBASE, Ovid PsycInfo, Ovid Cochrane Database of Systematic Reviews, Ovid Cochrane Central Register of Controlled Trials, and Scopus. Controlled vocabulary, supplemented with keywords, was used to search for the concept areas: suicide, sleep disorders, and psychiatric conditions. The database search strategy is available in Additional file 1. We also searched additional references from a recent systematic review [6].

### Study selection and data extraction

Reviewers working independently and in duplicate assessed each abstract for eligibility. Disagreements yielded an automatic inclusion into the following level of screening. Included studies were retrieved and full text screening commenced in duplicate as well. Disagreements at this level were resolved by discussion and consensus. Two reviewers working independently and in duplicate extracted baseline and outcome data and assessed the quality of each included study. A third reviewer compared the reviewers’ data and resolved inconsistencies by referring to the full text article.

Reviewers independently extracted study details from the full text articles, using a pilot-tested form. The following data were abstracted: study design, country, patient characteristics (sex, age), psychiatric diagnoses, sleep disturbances, and suicidal behaviors. We extracted numbers of patients with outcomes (suicidal behaviors) at the longest duration of complete follow-up.

### Assessment of study quality and publication bias

Using the Newcastle-Ottawa scale, we assessed the quality of included observational studies by determining outcome ascertainment, adjustment for confounders, and proportion of patients lost to follow-up as well as sample selection. We assessed potential publication bias by visual inspection of funnel plots and the Egger’s regression asymmetry tests.

### Statistical analysis

The outcome of interest was suicidal behaviors, including suicidal ideations, suicide attempts, and completed suicides. Outcomes were either dichotomized by the individual study or when presented as a scale, were converted to log transformed OR (logOR) based on the commonly used formula suggested by Borenstein et al. [10].

We pooled the logOR from all of the included studies using the DerSimonian & Laird random effects method with the estimate of heterogeneity from the Mantel-Haenszel model [11]. We conducted *a priori* planned subgroup analysis based on the type of suicidal behaviors (ideations, attempts, and completed suicides). We subsequently evaluated separately the different types of sleep disturbances (insomnia, hypersomnia, parasomnia, sleep-related breathing disorders, and mixed/unclear types) to further explore heterogeneity across studies. When one study reported outcomes for multiple subgroups, these outcomes were pooled separately in each subgroup. When multiple outcomes were reported in one subgroup, we chose the one that clearly defined and/or objectively measured.

We used the I^2^ statistic to measure heterogeneity across the studies. Statistical analyses were conducted using STATA version 12 (StataCorp, College Station, TX, USA) and R software version 3.0.1 (R Foundation for Statistical Computing, Vienna, Austria).

## Results

### Description and quality of included studies

**Figure 1:**
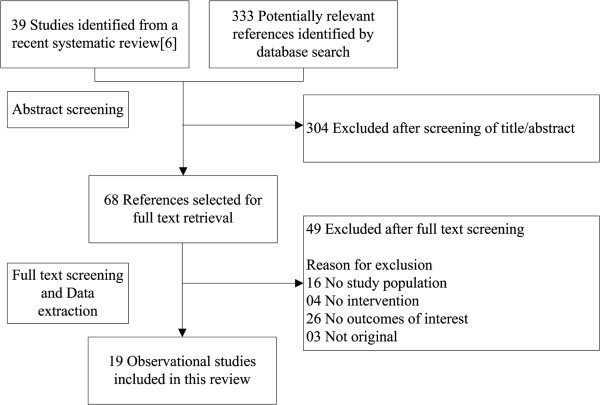
Flow diagram for selection of studies included in the systematic review.

Our search identified 372 candidate references (Figure 1). After deleting duplicated studies and excluding irrelevant studies, 19 studies met the inclusion criteria and were included in this analysis. The characteristics of these studies are summarized in Table 1. Overall, 104,436 patients were included in this review. The average age was 49.4 years (range, 17-79 years). Fifty-eight percent of the patients were female. The average length of follow-up in the prospective studies was 9.9 years (range, 3 days - 27 years). Of 19 studies, 13 (68.4%) reported outcomes in depressive patients. The other psychiatric diagnoses included were post-traumatic stress disorder (PTSD), panic disorder, schizophrenia, and anxiety. Eleven studies (57.9%) were prospective studies, four (21.1%) were cross-sectional, and three (15.87%) were retrospective.

**Table 1:** Basic characteristics of the included studies

**Author, year**	**Study design**	**Patients ( *n *)**	**Female (%)**	**Age (years)**	**Psychiatric diagnoses**	**Suicidal behaviors**	**Sleep disturbances**
Agargun, 1997 [7]	Retrospective	113	74%	Mean: 32.6 range: 18-70	Major depression	Ideation	Insomnia and hypersomnia assessed by SCID-I
Li, 2010 [8]	Cross-sectional	1,231	68.20%	Mean: 42.4, range 18-65	Major depression, Bipolar, Other	Attempt	Self-reported insomnia and recurrent nightmares assessed by a questionnaire
McGirr, 2007 [12]	Prospective	156	19.20%	Mean: 42.4	Major depression	Completed suicide	Insomnia and hypersomnia assessed by SCID-I
Nrugham, 2008 [13]	Prospective	2,464	50.80%	Mean age 13.7	Depressive symptom and disorder	Ideation, attempt	Insomnia, hypersomnia, non-restorative sleep assessed by K-SADS and MFQ
Paffenbarger, 1994 [14]	Prospective	21,582	0	Range: 35-74	Depressive disorder	Completed suicide	Self-reported insomnia assessed by a questionnaire
Pompili, 2009 [15]	Retrospective	40	10%	Mean: 40 Range: 23-76	Schizophrenia	Completed suicide	Insomnia defined as difficulty initiating or maintaining sleep, or non-restorative sleep causing impaired functioning or suffering that was long-lasting or demanded treatment and recorded from patients’ medical records
Sjostrom, 2009 [16]	Prospective	165	78%	Range: 18-69	Axis 1 DSM disorders	Attempt	Difficulties initiating sleep, problems maintaining sleep and early morning awakening assessed by USI
Agargun, 1997 [17]	Prospective	41	76%	Mean: 34.6 SD: 10.8	Major depression	SADS suicidality score	Subjective sleep quality, sleep latency, sleep duration, habitual sleep efficiency, sleep disturbance, use of sleeping medications, daytime dysfunction, and global severity measured by PSQI
Agargun, 1998 [18]	Prospective	63	76%	Mean: 34.1 SD: 10.8	Major depression	Ideation, attempts	Frequency of nightmares
Agargun, 1998 [19]	Unclear	67	73.10%	Mean: 31.9 SD: 8.8	Panic disorder	Ideation	Sleep panic and insomnia ascertained from SADS
Agargun, 2003 [20]	Prospective	26	61.50%	Unclear	Major depression	HDRS suicide scores	REM latency, REM%, REM periods measured by three nights of polysomnography
Chellappa, 2007 [21]	Cross-sectional	70	62.90%	Mean:: 40.5 SD: 12.54	Major depression	Ideation	Insomnia or excessive sleepiness assessed by the SHQ and ICSD
Krakow, 2000 [22]	Prospective	153	100%	Mean: 36.4 SD: 11.1	PTSD	Hamilton Depression and suicidality scores	SMD and SDB assessed by AASM 1997 and ASDA 1997
Agargun, 2007 [23]	Retrospective	100	52%	Mean: 32.1 SD: 10.7	Unipolar major depression	Attempt	Nightmares assessed by ICSD-R; insomnia assessed by the HDRS items 6, 7, and 8
Yoshimasu, 2006 [24]	Cross-sectional	231	57.10%	Mean: 36.3 SD: 28.5	Major depression	Ideation	Insomnia assessed by SDS, KMI, and patients’ three most painful complaints
Bjorngaard 2011 [25]	Prospective	74,977	51%	Mean: 37.9 SD: 16.0	Anxiety, depression	Completed suicide	Self-reported sleep difficulties assessed by a questionnaire
Li 2012 [26]	Prospective	419	81.80%	Mean: 44.6 SD: 10.4	Major depression	Ideation	Insomnia, nightmares, and frequency of sleep disturbances measured by a questionnaire
Krakow 2011 [27]	Cross-sectional	1,584	55%	Mean: 49.8 SD: 66.4	Depression	Attempt	Sleep disturbances measured by SMH, ISI DDNSI, FOSQ, and TMB-10
Fawcett, 1990 [1]	Prospective	954	58%	Mean: 38.1 Range: 17-79	Major affective disorder	Completed suicide	Insomnia assessed by SADS

AASM: American Academy of Sleep Medicine; ASDA: American Sleep Disorders Association; DDNSI: Disturbing Dream and Nightmare Severity Index; FOSQ: Functional Outcomes Sleep Questionnaire; HDRS: Hamilton Depression Rating Scale; ICSD: International Classification of Sleep Disorders diagnostic criteria for sleep disorders due to mood disorders; ICSD-R: International Classification of Sleep Disorders, revised; ISI: Insomnia Severity Index; KMI: Kyudai Medical Inventory; K-SADS: Kiddie-Schedule for Affective Disorders and Schizophrenia; MFQ: Mood and Feelings Questionnaire; PSQI: Pittsburgh Sleep Quality Index; PTSD: Post-traumatic stress disorder; REM: Rapid eye movement; SADS: Schedule for Affective Disorders and Schizophrenia; SCID-I: Structured Clinical Interview for DSM-IV-TR Axis I Disorders; SD: standard deviation; SDB: Sleep-disordered Breathing; SDS: Self-rating Depression Scale; SHQ: Sleep Habits questionnaire; SMD: Sleep-related Movement Disorders; SMH: Sleep Medicine History; TMB-10: Time Monitoring Behavior; USI: Uppsala Sleep Inventory.

**Figure 2:**
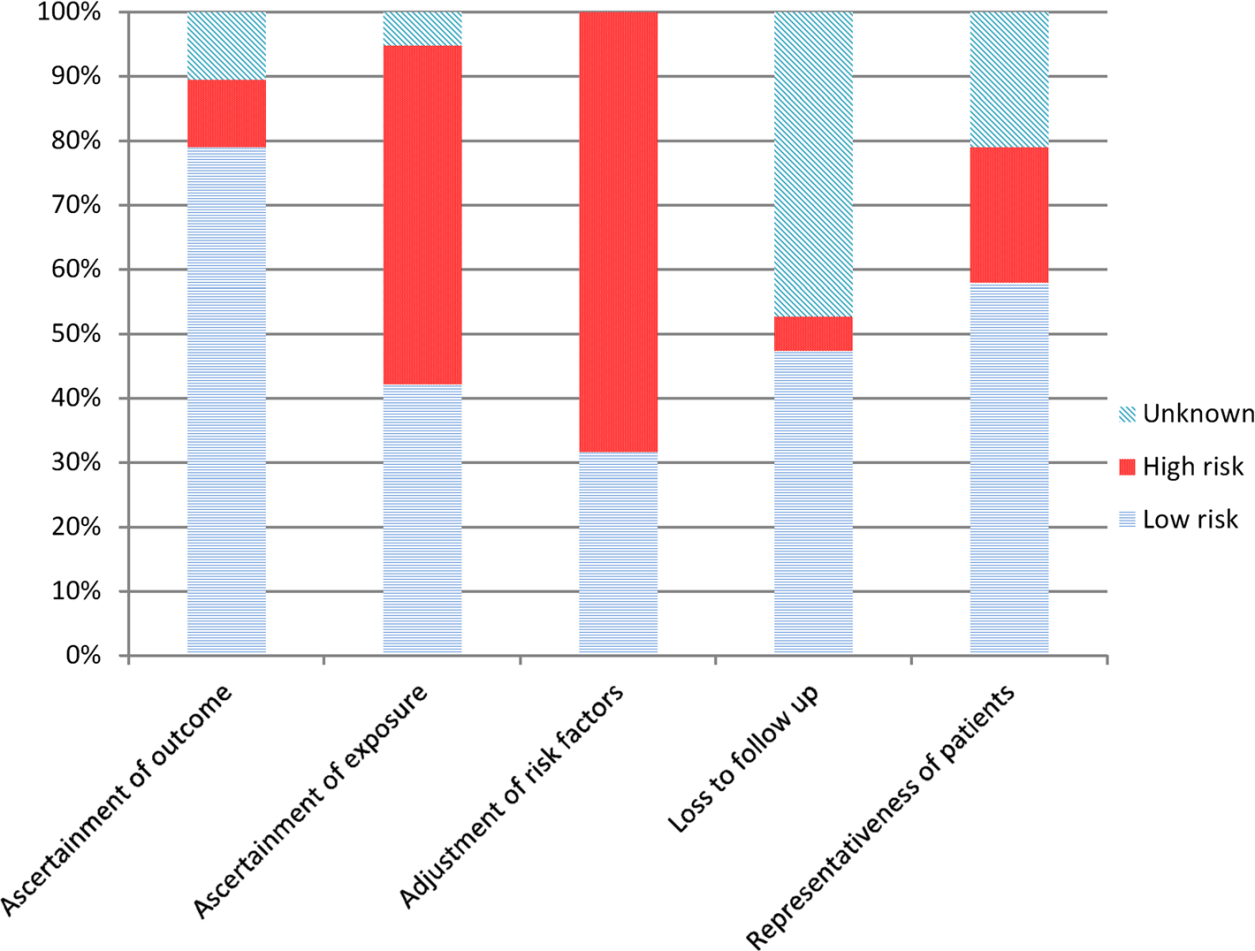
Quality assessment of the included studies.

Figure 2 shows the quality indicators of the included studies. We found variable quality across studies. Most (68.4%) of the studies did not adjust for risk factors. However, 15 studies reported low risk of bias for the ascertainment of outcomes. Using the Egger regression asymmetry test and visual inspection of the funnel plot, we found potential publication bias in this body of evidence. Across the included studies, substantial heterogeneity was observed in most of the pooled outcomes (>50%) [28]. In summary, the risk of bias is moderate to high due to potential publication bias and substantial heterogeneity.

**Figure 3:**
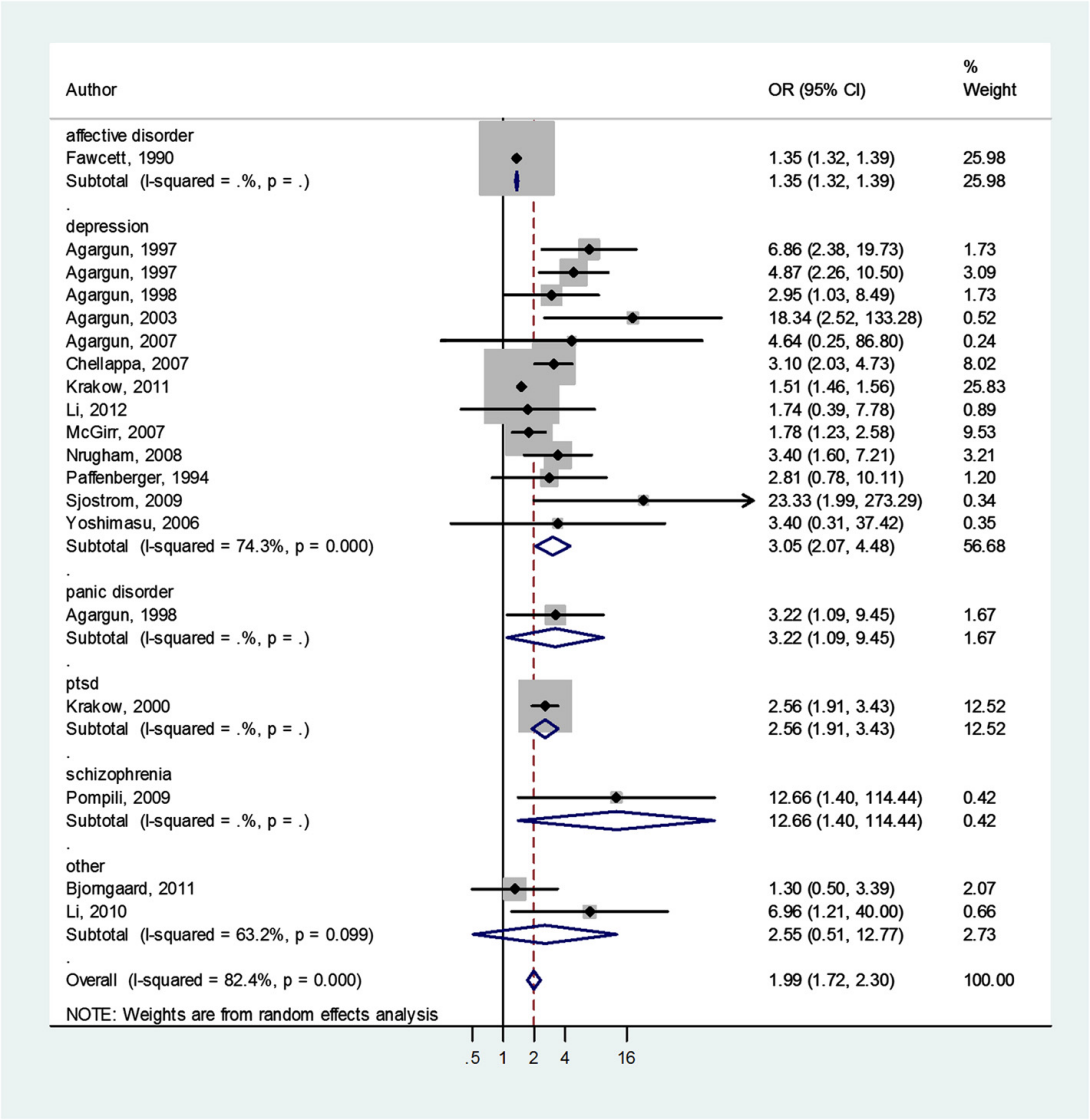
Forest plot of the association between sleep disturbances and suicidal behaviors in patients with psychiatric diagnoses.

Figure 3 shows the pooled odds ratios (ORs) from all of the included studies. Compared to those without sleep disturbances, patients with psychiatric diagnoses and co-morbid sleep disturbances were significantly more likely to report suicidal behaviors (OR = 1.99, 95% CI 1.72, 2.30, *P* <0.001). Regarding to specific psychiatric conditions, we found strong association between suicidal behaviors and sleep disturbances in depression (OR = 3.05, 95% CI 2.07, 4.48, *P* <0.001), PTSD (OR = 2.56, 95% CI 1.91, 3.43, *P* <0.001), panic disorders (OR = 3.22, 95% CI 1.09, 9.45, *P* = 0.03), and schizophrenia (OR = 12.66, 95% CI 1.40, 114.44, *P* = 0.02). Table 2 shows the pooled OR for different suicidal behaviors. Sleep disturbances were also significantly associated with suicide ideations, suicide attempts, and completed suicides. Subgroup analysis based on the type of sleep disturbance showed that insomnia, parasomnia, and sleep-related breathing disorders were significantly associated with increased risk of suicidal behaviors (Table 3). The only exception was hypersomnias (OR = 1.91, 95% CI: 0.60, 6.06, *P* = 0.27).

**Table 2:** Subgroup analysis based on suicidal behaviors

**Suicidal behaviors**	**OR**	**95% CI**	***P *** **value**	**I**^**2**^
Ideation	2.69	1.62, 4.48	<0.001	73.5%
Attempt	4.36	2.28, 8.33	<0.001	0.0%
Completed suicide	1.59	1.17, 2.17	<0.01	45.0%

**Table 3:** Subgroup analysis based on sleep disturbance type

**Sleep disturbance type**	**Studies ( *n *)**	**OR**	**95% CI**	***P *** **value**	**I**^**2**^
Insomnia	11	2.66	1.74, 4.07	<0.001	75.4%
Hypersomnia	3	1.91	0.60, 6.06	0.27	85.8%
Parasomnia	6	4.69	2.58, 8.51	<0.001	0.0%
Sleep-related breathing disorder	1	2.56	1.91, 3.43	<0.001	n/a
Other/unclear	7	4.16	1.96, 8.81	<0.001	81.7%

Sleep-related breathing disorder (SBD): only one study (Krakow, 2000) reported SBD and no objective test was used to confirm the diagnosis.

## Discussion

We conducted a systematic review and meta-analysis to investigate the association between the presence of sleep disturbances and risk of suicidal behaviors in patients with psychiatric diagnoses. Nineteen studies with 104,436 patients were included in the analysis. We found significantly increased risk of suicidal behaviors among patients with sleep disturbances and psychiatric diagnoses compared to those without sleep disturbances.

Nevertheless, the causal role or mechanism of sleep disturbances in suicidal behavior remains unclear. It is suggested that sleep disturbances exacerbates psychological distress [4,5], rendering psychiatric patients more vulnerable to suicidal behavior as a way to reduce or escape from such distress. There is also evidence that fatigue subsequent to sleep problems may impair problem solving and decrease emotion regulation increasing one’s risk for suicidal behavior through vulnerability to impulsive behavior [29]. Other possibilities may include changes in sleep architecture that render one vulnerable to suicide. Certainly, future research is necessary to examine the specific mechanism of vulnerability because such knowledge may lead to an effective intervention. Research should establish whether there are specific problems in sleep or sleep architecture that promotes vulnerability, and should determine whether there are specific sleep profiles (for example, nightmares, insomnia, early morning awakening) that may discriminate between individuals who are at risk to suicide behaviors and those who actually complete suicide.

### Clinical implications

Given the findings of this study, the presence of sleep disturbances in individuals with psychiatric illness may trigger the need for further evaluation of increased risk for suicide. In particular, clinicians evaluating psychiatric patients who report acute changes in sleep should be aware that this may represent a specific vulnerability for suicide, and a careful suicide risk assessment may be warranted. Correspondingly, a comprehensive suicide risk assessment may include an evaluation of sleep quality and maintenance. Screening measures such as the Pittsburgh Sleep Quality Index (PSQI) [30], which assesses sleep quality, efficiency, duration, disturbances, and daytime dysfunction, and has good reliability and validity in detecting sleep problems, may assist in this effort. A suicide risk assessment that includes evaluation of sleep disturbances may not only add to the estimation of risk, but it may also provide a potential target for intervention. This is particularly promising given that many of the previously established risk factors are person variables such as age, type of psychiatric disorder, and family history of suicide attempts, and thereby not amenable to intervention.

In terms of intervention, there is considerable evidence for the efficacy of Cognitive Behavioral Therapy for Insomnia (CBT-I) with large effect sizes found in meta-analytic studies [31]. Not only is CBT-I effective for treating primary sleep disorders, research examining CBT-I in patients with major depression and co-morbid insomnia found that the treatment led to improvements in both disorders [32]. Although CBT-I is considered to be a first-line treatment for insomnia [33], pharmacological treatments may also be given consideration for insomnia and other sleep problems. However, caution should be taken as studies found sedative-hypnotics were associated with increased suicidal behaviors [34-37]. A large cohort study demonstrated that receiving hypnotic prescriptions was associated with greater than three-fold increased hazards of death even when prescribed <18 pills/year. This association held in separate analyses for several commonly used hypnotics and for newer shorter-acting drugs [38].

### Limitations and strengths

Strengths of the study include the comprehensive literature search, application of bias protection measures in the study selection, and careful evaluation of methodological quality. There are important limitations. Observational studies are subject to high risk of bias due to potential outcome confounding. Substantial heterogeneity was observed across the studies. Publication bias may have also affected our results. At last, we observed substantial variations of how the included studies define sleep disturbances and suicidal behaviors, and of how these studies measure them. All of the above may affect our findings. Based on the Grading of Recommendations Assessment, Development and Evaluation (GRADE) methodology, the strength of the current evidence is low due to potential publication bias and heterogeneity [39].

## Conclusions

The current evidence and the results of this systematic review and meta-analysis suggest that in patients with psychiatric diagnoses, sleep disturbances are associated with the increased risk of suicidal behaviors.

## Abbreviations

95% CI: 95% Confidence interval; CBT-I: Cognitive behavioral therapy for insomnia; GRADE: Grading of recommendations assessment development and evaluation; OR: Odds ratio; PRISMA: Preferred Reporting Items for Systematic Reviews and Meta-analyses; PSQI: Pittsburgh Sleep uality Index; PTSD: Post-traumatic stress disorder.

## Competing interests

The authors declare that they have no competing interests.

## Authors’ contributions

SM carried out study design, study screening, data extraction, quality appraisal, and drafted the manuscript. AK participated in study design, study screening, data extraction, and critically revised the manuscript. LS conducted data extraction, drafted, and critically revised the manuscript. LP designed the search strategy and revised the manuscript. ZW carried out study design, study screening, data extraction, quality appraisal, data analysis, and drafted the manuscript. KB participated in data extraction, quality appraisal, and critically revised the manuscript. MHM carried out study design, advised on all methodological issues, drafted, and critically revised the manuscript. SM and ZW had equal contributions to the manuscript. All authors approved the final version of this manuscript.

## Supplementary Material

Additional file 1 Database search strategy. Click here for file

## References

[B1] Fawcett J Scheftner WA Fogg L Clark DC Young MA Hedeker D Gibbons R Time-related predictors of suicide in major affective disorder Am J Psychiatry 1990 147 1189 1194 2104515 10.1176/ajp.147.9.1189

[B2] Mann JJ Waternaux C Haas GL Malone KM Toward a clinical model of suicidal behavior in psychiatric patients Am J Psychiatry 1999 156 181 189 9989552 10.1176/ajp.156.2.181

[B3] Goldstein RB Black DW Nasrallah A Winokur G The prediction of suicide. Sensitivity, specificity, and predictive value of a multivariate model applied to suicide among 1906 patients with affective disorders Arch Gen Psychiatry 1991 48 418 422 10.1001/archpsyc.1991.01810290030004 2021294

[B4] Breslau N Roth T Rosenthal L Andreski P Sleep disturbance and psychiatric disorders: a longitudinal epidemiological study of young adults Biol Psychiatry 1996 39 411 418 10.1016/0006-3223(95)00188-3 8679786

[B5] Ford DE Kamerow DB Epidemiologic study of sleep disturbances and psychiatric disorders. An opportunity for prevention? JAMA 1989 262 1479 1484 10.1001/jama.1989.03430110069030 2769898

[B6] Pigeon WR Pinquart M Conner K Meta-analysis of sleep disturbance and suicidal thoughts and behaviors J Clin Psychiatry 2012 73 e1160 e1167 10.4088/JCP.11r07586 23059158

[B7] Agargun MY Kara H Solmaz M Sleep disturbances and suicidal behavior in patients with major depression J Clin Psychiatry 1997 58 249 251 10.4088/JCP.v58n0602 9228889

[B8] Li SX Lam SP Yu MW Zhang J Wing YK Nocturnal sleep disturbances as a predictor of suicide attempts among psychiatric outpatients: a clinical, epidemiologic, prospective study J Clin Psychiatry 2010 71 1440 1446 10.4088/JCP.09m05661gry 21114949

[B9] Moher D Liberati A Tetzlaff J Altman DG Preferred reporting items for systematic reviews and meta-analyses: the PRISMA statement Ann Intern Med 2009 151 264 269 W264 10.7326/0003-4819-151-4-200908180-00135 19622511

[B10] Borenstein M Cooper H Hedges L Valentine J Cooper H, Hedges LV, Valentine JC Effect sizes for continuous data The Handbook of Research Synthesis and Meta-Analysis 2009 New York, NY: Russell Sage 221 235

[B11] DerSimonian R Laird N Meta-analysis in clinical trials Control Clin Trials 1986 7 177 188 10.1016/0197-2456(86)90046-2 3802833

[B12] McGirr A Renaud J Seguin M Alda M Benkelfat C Lesage A Turecki G An examination of DSM-IV depressive symptoms and risk for suicide completion in major depressive disorder: a psychological autopsy study J Affect Disord 2007 97 203 209 10.1016/j.jad.2006.06.016 16854469

[B13] Nrugham L Larsson B Sund AM Specific depressive symptoms and disorders as associates and predictors of suicidal acts across adolescence J Affect Disord 2008 111 83 93 10.1016/j.jad.2008.02.010 18395267

[B14] Paffenbarger RS Jr Lee IM Leung R Physical activity and personal characteristics associated with depression and suicide in American college men Acta Psychiatr Scand Suppl 1994 377 16 22 8053361 10.1111/j.1600-0447.1994.tb05796.x

[B15] Pompili M Lester D Grispini A Innamorati M Calandro F Iliceto P De Pisa E Tatarelli R Girardi P Completed suicide in schizophrenia: evidence from a case–control study Psychiatry Res 2009 167 251 257 10.1016/j.psychres.2008.03.018 19395048

[B16] Sjostrom N Hetta J Waern M Persistent nightmares are associated with repeat suicide attempt: a prospective study Psychiatry Res 2009 170 208 211 10.1016/j.psychres.2008.09.006 19900715

[B17] Agargun MY Kara H Solmaz M Subjective sleep quality and suicidality in patients with major depression J Psychiatr Res 1997 31 377 381 10.1016/S0022-3956(96)00037-4 9306295

[B18] Agargun MY Cilli AS Kara H Tarhan N Kincir F Oz H Repetitive and frightening dreams and suicidal behavior in patients with major depression Compr Psychiatry 1998 39 198 202 10.1016/S0010-440X(98)90060-8 9675503

[B19] Agargun MY Kara H Recurrent sleep panic, insomnia, and suicidal behavior in patients with panic disorder Compr Psychiatry 1998 39 149 151 10.1016/S0010-440X(98)90074-8 9606581

[B20] Agargun MY Cartwright R REM sleep, dream variables and suicidality in depressed patients Psychiatry Res 2003 119 33 39 10.1016/S0165-1781(03)00111-2 12860358

[B21] Chellappa SL Araujo JF Sleep disorders and suicidal ideation in patients with depressive disorder Psychiatry Res 2007 153 131 136 10.1016/j.psychres.2006.05.007 17658614

[B22] Krakow B Artar A Warner TD Melendrez D Johnston L Hollifield M Germain A Koss M Sleep disorder, depression, and suicidality in female sexual assault survivors Crisis 2000 21 163 170 11419527 10.1027//0227-5910.21.4.163

[B23] Agargun MY Besiroglu L Cilli AS Gulec M Aydin A Inci R Selvi Y Nightmares, suicide attempts, and melancholic features in patients with unipolar major depression J Affect Disord 2007 98 267 270 10.1016/j.jad.2006.08.005 16938351

[B24] Yoshimasu K Sugahara H Akamine M Kondo T Fujisawa K Tokunaga S Kiyohara C Miyashita K Kubo C Sleep disorders and suicidal ideation in Japanese patients visiting a psychosomatic clinic in a university hospital Sleep and Biological Rhythms 2006 4 137 143 10.1111/j.1479-8425.2006.00209.x

[B25] Bjorngaard JH Bjerkeset O Romundstad P Gunnell D Sleeping problems and suicide in 75,000 Norwegian adults: a 20 year follow-up of the HUNT I study Sleep 2011 34 1155 1159 21886352 10.5665/SLEEP.1228 PMC3157656

[B26] Li SX Lam SP Chan JWY Yu MWM Wing Y-K Residual sleep disturbances in patients remitted from major depressive disorder: a 4-year naturalistic follow-up study Sleep 2012 35 1153 1161 22851811 10.5665/sleep.2008 PMC3397819

[B27] Krakow B Ribeiro JD Ulibarri VA Krakow J Joiner TE Jr Sleep disturbances and suicidal ideation in sleep medical center patients J Affect Disord 2011 131 422 427 10.1016/j.jad.2010.12.001 21211850

[B28] Higgins JP Thompson SG Deeks JJ Altman DG Measuring inconsistency in meta-analyses BMJ 2003 327 557 560 10.1136/bmj.327.7414.557 12958120 PMC192859

[B29] Anderson C Platten CR Sleep deprivation lowers inhibition and enhances impulsivity to negative stimuli Behav Brain Res 2011 217 463 466 10.1016/j.bbr.2010.09.020 20888369

[B30] Buysse DJ Reynolds CF 3rd Monk TH Berman SR Kupfer DJ The Pittsburgh Sleep Quality Index: a new instrument for psychiatric practice and research Psychiatry Res 1989 28 193 213 10.1016/0165-1781(89)90047-4 2748771

[B31] Morin CM Culbert JP Schwartz SM Nonpharmacological interventions for insomnia: a meta-analysis of treatment efficacy Am J Psychiatry 1994 151 1172 1180 8037252 10.1176/ajp.151.8.1172

[B32] Manber R Edinger JD Gress JL San Pedro-Salcedo MG Kuo TF Kalista T Cognitive behavioral therapy for insomnia enhances depression outcome in patients with comorbid major depressive disorder and insomnia Sleep 2008 31 489 495 18457236 10.1093/sleep/31.4.489 PMC2279754

[B33] Chesson AL Jr Anderson WM Littner M Davila D Hartse K Johnson S Wise M Rafecas J Practice parameters for the nonpharmacologic treatment of chronic insomnia, An American Academy of Sleep Medicine report. Standards of Practice Committee of the American Academy of Sleep Medicine Sleep 1999 22 1128 1133 10617175 10.1093/sleep/22.8.1128

[B34] Allgulander C Ljungberg L Fisher LD Long-term prognosis in addiction on sedative and hypnotic drugs analyzed with the Cox regression model Acta Psychiatr Scand 1987 75 521 531 10.1111/j.1600-0447.1987.tb02828.x 3604738

[B35] Carlsten A Waern M Are sedatives and hypnotics associated with increased suicide risk of suicide in the elderly? BMC Geriatr 2009 9 20 10.1186/1471-2318-9-20 19497093 PMC2695460

[B36] Rod NH Vahtera J Westerlund H Kivimaki M Zins M Goldberg M Lange T Sleep disturbances and cause-specific mortality: results from the GAZEL cohort study Am J Epidemiol 2011 173 300 309 10.1093/aje/kwq371 21193534 PMC3105272

[B37] Belleville G Mortality hazard associated with anxiolytic and hypnotic drug use in the National Population Health Survey Can J Psychiatry 2010 55 558 567 20840803 10.1177/070674371005500904

[B38] Kripke DF Langer RD Kline LE Hypnotics’ association with mortality or cancer: a matched cohort study BMJ Open 2012 2 e000850 22371848 10.1136/bmjopen-2012-000850 PMC3293137

[B39] Balshem H Helfand M Schunemann HJ Oxman AD Kunz R Brozek J Vist GE Falck-Ytter Y Meerpohl J Norris S Guyatt GH GRADE guidelines: 3. Rating the quality of evidence J Clin Epidemiol 2011 64 401 406 10.1016/j.jclinepi.2010.07.015 21208779

