# Supplementary material for: The association between sleep disturbances and suicidal behaviors in patients with psychiatric diagnoses: a systematic review and meta-analysis
Source: Syst Rev. 2014 Feb 25;3:18. doi: 10.1186/2046-4053-3-18 (PMC3945796; doi:10.1186/2046-4053-3-18)
Supplement: Additional file 1 — Database search strategy. [file 2046-4053-3-18-S1.pdf]

Additional file 1

<u>Ovid</u>

Database(s): Embase 1988 to June 28 2013, Ovid MEDLINE(R) In-Process & Other Non-Indexed Citations and Ovid MEDLINE(R) 1948 to June 28 2013, PsycINFO 1987 to June 28 2013, EBM Reviews - Cochrane Central Register of Controlled Trials 2nd Quarter 2013, EBM Reviews - Cochrane Database of Systematic Reviews 2005 to June 28 2013.

Search Strategy:

#	Searches
1	exp Suicide/
2	suicid*.mp.
3	1 or 2
4	exp Sleep Disorders/
5	((sleep adj2 disorder*) or "sleeper syndrome*" or "subwakefullness syndrome*" or "sleep-related neurogenic tachypnea*" or dyssomnia* or parasomnia* or insomnia* or "sleep initiation dysfunction" or (sleep adj3 (initiat* or maintenance or maintain*) adj3 (disorder* or dysfunction)) or "early awakening" or sleeplessness or "sleep deprivation" or "jet lag" or somnolence or hypersomnolence or "kleine-levin syndrome" or narcolepsy or cataplexy or "nocturnal myoclonus syndrome" or "restless legs syndrome" or "restless leg syndrome" or "sleep apnea" or "obesity hypoventilation syndrome" or "nocturnal paroxysmal dystonia" or

("rem sleep" adj2 disorder*) or "sleep paralysis" or "night terror*" or somnambulism or "sleep bruxism" or "sleep-wake transistion disorder*" or "chronobiology disorder*" or "abnormal dreaming" or "sleep myoclonus" or somnolence or "excessive dreaming" or "exploding head syndrome" or hypersomnia or "hypnagogic hallucination*" or "hypnic headache*" or nightmare* or paroniria or "periodic limb movement disorder*" or "sleep arousal disorder*" or "sleep disordered breathing" or "sleep walking" or sopor or "vivid dream*").mp. [mp=ti, ab, sh, hw, tn, ot, dm, mf, dv, kw, ps, rs, nm, ui, tc, id, tm, tx, ct]

6 4 or 5

7 exp cohort analysis/

8 exp case control study/

9 exp Cohort Studies/

10 exp case-control studies/

11 ((cohort or concurrent or incidence or "follow-up" or longitudinal or retrospective or prospective) adj (study or studies or analysis or analyses)).mp. [mp=ti, ab, sh, hw, tn, ot, dm, mf, dv, kw, ps, rs, nm, ui, tc, id, tm, tx, ct]

12 (("case-control" or "case-referrent" or "case-compeer" or "case-comparison" or "case-base") adj (study or studies or analysis or analyses)).mp. [mp=ti, ab, sh, hw, tn, ot, dm, mf, dv, kw, ps, rs, nm, ui, tc, id, tm, tx, ct]

13 or/7-12

14 3 and 6 and 13

15 remove duplicates from 14

16 limit 15 to (book or book series or editorial or erratum or letter or trade journal or addresses or autobiography or bibliography or biography or dictionary or directory or duplicate publication or in vitro or interactive tutorial or interview or lectures or legal cases or legislation or news or newspaper article or overall or patient education handout or periodical index or portraits or webcasts or "0200 book" or "0240 authored book" or "0280 edited book" or "0300 encyclopedia") [Limit not valid in Embase,Ovid MEDLINE(R),Ovid MEDLINE(R) In-Process,PsycINFO,CCTR,CDSR; records were retained]

17 from 16 keep 1-5

18 15 not 17

Scopus

1 TITLE-ABS-KEY(suicid*)

2 TITLE-ABS-KEY((sleep W/2 disorder*) or "sleeper syndrome*" or "subwakefullness syndrome*" or "sleep-related neurogenic tachypnea*" or dyssomnia* or parasomnia* or insomnia* or "sleep initiation dysfunction" or "early awakening" or sleeplessness or "sleep deprivation" or "jet lag" or somnolence or hypersomnolence or "kleine-levin syndrome" or narcolepsy or cataplexy or "nocturnal myoclonus syndrome" or "restless legs syndrome" or "restless leg syndrome" or "sleep apnea" or "obesity hypoventilation syndrome" or "nocturnal paroxysmal dystonia" or ("rem sleep" W/2 disorder*) or "sleep paralysis" or "night terror*" or somnambulism or "sleep bruxism" or "sleep-wake transistion disorder*" or "chronobiology disorder*" or "abnormal dreaming" or "sleep myoclonus" or somnolence or "excessive dreaming" or "exploding head syndrome" or hypersomnia or "hypnagogic hallucination*" or "hypnic headache*" or nightmare* or paroniria or "periodic limb movement disorder*" or "sleep arousal disorder*" or "sleep disordered breathing" or "sleep walking" or sopor or "vivid dream*" or (sleep W/3 initiat* W/3 disorder*) or (sleep W/3 initiat* W/3 dysfunction) or (sleep W/3 maintenance W/3 disorder*) or (sleep W/3 maintenance W/3 dysfunction) or (sleep W/3 maintain* W/3 disorder*) or (sleep W/3 maintain* W/3 dysfunction))

3 TITLE-ABS-KEY((cohort W/1 study) or (cohort W/1 studies) or (cohort W/1 analysis) or (cohort W/1 analyses) or (concurrent W/1 study) or (concurrent W/1 studies) or (concurrent W/1 analysis) or (concurrent W/1 analyses) or (incidence W/1 study) or (incidence W/1 studies) or (incidence W/1 analysis) or (incidence W/1 analyses) or ("follow-up" W/1 study) or ("follow-up" W/1 studies) or ("follow-up" W/1 analysis) or

("follow-up" W/1 analyses) or (longitudinal W/1 study) or (longitudinal W/1 studies) or (longitudinal W/1 analysis) or (longitudinal W/1 analyses) or (retrospective W/1 study) or (retrospective W/1 studies) or (retrospective W/1 analysis) or (retrospective W/1 analyses) or (prospective W/1 study) or (prospective W/1 studies) or (prospective W/1 analysis) or (prospective W/1 analyses) or ("case-control" W/1 study) or ("case-control" W/1 studies) or ("case-control" W/1 analysis) or ("case-control" W/1 analyses) or ("case-referrent" W/1 study) or ("case-referrent" W/1 studies) or ("case-referrent" W/1 analysis) or ("case-referrent" W/1 analyses) or ("case-compeer" W/1 study) or ("case-compeer" W/1 studies) or ("case-compeer" W/1 analysis) or ("case-compeer" W/1 analyses) or ("case-comparison" W/1 study) or ("case-comparison" W/1 studies) or ("case-comparison" W/1 analysis) or ("case-comparison" W/1 analyses) or ("case-base" W/1 study) or ("case-base" W/1 studies) or ("case-base" W/1 analysis) or ("case-base" W/1 analyses))

4 1 and 2 and 3

5 PMID(0*) OR PMID(1*) OR PMID(2*) OR PMID(3*) OR PMID(4*) OR PMID(5*) OR PMID(6*) OR PMID(7*) OR PMID(8*) OR PMID(9*)

6 4 and not 5

7 DOCTYPE(le) OR DOCTYPE(ed) OR DOCTYPE(bk) OR DOCTYPE(er) OR DOCTYPE(no) OR DOCTYPE(sh)

8 6 and not 7
